# Polyurethane Wood Adhesives Prepared from Modified Polysaccharides

**DOI:** 10.3390/polym14030539

**Published:** 2022-01-28

**Authors:** Reza Hosseinpourpia, Arantxa Eceiza, Stergios Adamopoulos

**Affiliations:** 1 Department of Forestry and Wood Technology, Linnaeus University, Lückligs Plats 1, 35195 Växjö, Sweden; 2 Materials + Technologies’ Group, Department of Chemical & Environmental Engineering, Polytechnic College of San Sebastian, University of the Basque Country UPV/EHU, Pza. Europa 1, 20018 Donostia-San Sebastián, Spain; arantxa.eceiza@ehu.eus; 3 Department of Forest Biomaterials and Technology, Swedish University of Agricultural Sciences, Vallvägen 9C, 75007 Uppsala, Sweden; stergios.adamopoulos@slu.se

**Keywords:** polyurethane adhesive, wheat starch, pMDI, isocyanate chemistry, wood adhesive

## Abstract

This study investigated the performance of polyurethane adhesives prepared with various combinations of wheat starch that had been modified by isophorone diisocyanate (MS), two polyol types (1,3-propanediol (PD) and glycerol (Gly)), native wheat starch (NS), and 4,4′-diphenylmethane diisocyanate (pMDI) at a NCO:OH weight ratio of 1:1. Two more adhesives were also synthesized with NS, PD, or Gly and pMDI blends and served as controls. The thermal behavior of the adhesives before and after the curing process, as well as their rheological performance and lap shear strength, were analyzed. Differential scanning calorimetry (DSC) showed a reduction in curing temperature and heat by adding MS. The thermal stability of the cured adhesives was slightly increased by MS addition. The viscosity of the adhesives that contained MS substantially increased at a linear ascendant ramp of shear, while the controls exhibited relatively low viscosity during the whole shear rate spectrum from 0.1 to 100 s^−1^. The tensile shear strength of wood veneers was also significantly increased by the incorporation of MS under both dry and wet measuring conditions. The maximum dry shear strength was obtained for the adhesive with Gly polyol and a higher content of MS and was comparable to the control adhesive with pMDI.

## 1. Introduction

The global wood adhesives and binder market is increasing tremendously and is expected to exceed USD 21 billion by 2024 [1]. A major part of the adhesives used today is formaldehyde-based, such as urea-formaldehyde, phenol-formaldehyde, and melamine-formaldehyde. Besides the hydrolyzation issue of formaldehyde-based adhesives under warm and humid conditions, increasing concerns related to formaldehyde emissions, as a potential hazard to the environment and human health, have forced the wood industry to find alternative, formaldehyde-free adhesives [2]. Polyurethane wood adhesive (PUWA) offers non-formaldehyde emitting solutions, and it has been introduced as an alternative adhesive in the wood panel industry [3]. PUWAs are generally prepared by the reaction between hydroxyl and isocyanate groups to form urethane linkages [4] and demonstrate strong adhesion and good durability. However, they are not considered a sustainable solution, as most of them are petroleum derived.

Due to the limited variety of isocyanate, the majority of the published research on renewable raw materials for the preparation of PUWAs focuses on the methods used to develop polyols from renewable resources [5,6] such as vegetable oils [7,8,9,10,11], polysaccharides [12,13], and lignocellulosic biomass [4,13,14]. Starch, as the main reserve source and energy storage of plants, has been extensively used in the food, textile, pharmaceutical, paper, and biofuel sectors [15]. However, the application of starch as a sole wood adhesive is limited due to high viscosity, poor storage stability, and insufficient bonding capacity [16]. The chemical modification of starch improves its hydrophobicity, mechanical performance, thermal stability, compatibility with other polymers, and bonding quality. Valodkar and Thakore (2010) quoted that the modification of starch nanoparticles with 1,4-hexamethylene diisocyanate (HMDI) enhanced the electrical conductivity of the polyether–polyurethane matrix due to the formation of crosslinked hydrophobic nanoparticles [17]. The acetylation of cassava starch by propionic anhydride also increased the mechanical strength of the polyurethane matrices [18]. To the best of our knowledge, there are limited studies on the utilization of starch in PUWA [12,19], where starch mainly acts as a polyol or a reinforcement in the adhesive system. Desai and coworkers (2003) prepared PUWAs using polyester polyols from potato starch, natural oils, e.g., castor and argemone oils, and toluene 2,4-diisocyanate (TDI) at various levels of polyols with regard to their hydroxyl values [13]. The authors reported superior performance of the renewable polyols in PUWA as compared with the synthetic ones [13]. Isocyanate prepolymer-starch adhesive was prepared by the blending of acid-hydrolyzed and then oxidized corn starch and different levels of carboxymethyl cellulose (CMC) with polymethylene polyphenyl polyisocyanate (PAPI) prepolymer. The thermal stability and bonding strength of the adhesive were improved by increasing the content of PAPI and CMC [20]. The modification of starch with blocked isocyanate also considerably enhanced the shear strength of plywood panels under dry measuring conditions [21].

The functionalization of pea starch and dextrin with isophorone diisocyanate (IPDI) monomer enhanced the thermal stability and hydrophobicity of starch and dextrin polymers as compared to unmodified ones [15]. Recently, native wheat starch modified with IPDI (at a NCO:OH ratio of 6:1) was used for the preparation of polyurethane films with combinations of polyols and polymeric diphenylmethane diisocyanate (pMDI). Various types of polyurethane films were prepared using native wheat starch and two polyol types (1,3-propanediol (PD) and glycerol (Gly)) as hydroxyl group (–OH) donors and IPDI modified starch and pMDI as isocyanate group (–NCO) donors at a NCO:OH weight ratio of 1:1. The polyurethane films were then studied for their chemical structure, thermal behavior, thermomechanical and flexural properties, and microstructural characteristics. The results exhibited a significant contribution of modified starch in the improvement of the mechanical, thermal, thermomechanical, and microstructural performances of polyurethane films [22]. This study was built upon the previous promising results with the aim to evaluate PUWAs based on IPDI-functionalized wheat starch by means of curing behavior, rheological performance, and lap shear strength of bonded wood veneers under dry and wet measuring conditions.

## 2. Materials and Methods

### 2.1. Materials

Native wheat starch (NS) was kindly provided by Lantmännen (Stockholm, Sweden). The modified starch, MS, was obtained from our previous work [22] by the chemical modification of NS with isophorone diisocyanate IPDI (>99.5%, Desmodur I^®^, kindly provided by Covestro, Leverkusen, Germany), and a modification efficiency of 0.49. Polymeric diphenylmethane diisocyanate pMDI (>99.5%, Desmodur 44V20L) was kindly provided by Covestro (Leverkusen, Germany). Two polyols, 1,3-propanediol (PD) (bio-PDO, Susterra^®^, DuPont Tate & Lyle Bio Products Co., Loudon, TN, USA) and glycerol (Gly) (vegetable origin for analysis EMSURE^®^ ACS, Reag. Ph Eur, Sigma Aldrich, Saint Louis, MO, USA) were used to prepare the adhesives.

### 2.2. Adhesive Preparation

Various combinations were used for the preparation of the adhesives according to Hosseinpourpia et al. (2021). In brief, the isocyanate (–NCO) groups were delivered by pMDI and MS, while NS and the two polyols (PD and Gly) supplied the hydroxyl (–OH) groups. The mixing levels (wt.) of each component were calculated based on their equivalent number, as determined by the content of –NCO and –OH groups according to ASTM D 2572-97 and ASTM D 4274-05 standards, respectively. All adhesives were prepared at an equivalent NCO:OH ratio of 1:1 according to Hosseinpourpia et al. [22], as shown in Table 1. NS was first magnetically stirred with polyol (PD or Gly) for 3 min at room temperature, and then, MS and pMDI were added to the mixture and allowed to magnetically stir for another 2 min. Two adhesives were also prepared for each polyol without MS and served as controls.

### 2.3. Differential Scanning Calorimetry (DSC) Analysis

The curing behavior of the adhesives directly after blending was evaluated by a DSC analyzer (Mettler Toledo DSC3+ equipment, Columbus, OH, USA), from −40 to 220 °C at a heating rate of 10 °C min^−1^ under a nitrogen flow of 10 mL min^−1^. For the determination of glass transition temperature (Tg), similar sets of adhesives were prepared and cured at 160 °C for 30 min.

### 2.4. Thermogravimetric Analysis

The thermal stability of cured adhesives (160 °C for 30 min) was analyzed using Q500 TA equipment (New Castle, DE, USA). Around 5 mg of each sample was heated from 25 °C to 750 °C at a rate of 10 °C min^−1^, under a nitrogen atmosphere.

### 2.5. Rheological Performance

Rheological measurements were carried out at 20 °C using a HAAKE viscotester IQ from Thermo Scientific™ (Waltham, MA, USA) with parallel plate (35 mm) geometry and a gap of about 0.5 mm. The viscosity of the uncured adhesive formulations containing different levels of bio-based and synthetic polymers was determined at a logarithmic ascendant ramp for a shear rate from 0.1 to 100 s^−1^.

### 2.6. Shear Strength Test

The bonding quality of the adhesives was evaluated by the tensile shear strength test. Birch wood veneers measuring 70 × 25 × 1.5 mm^3^ (L × W × T) were conditioned at 20 °C and 65% relative humidity (RH) for at least seven days before gluing. The extremities of two veneer strips (625 mm^2^ overlapped area) were bonded with a thin line of glue (360 g adhesive.m^−2^). The bonded veneer strips were hot-pressed at 160 °C and 0.7 MPa for 30 min. Ten samples were prepared with each adhesive and conditioned at 20 °C and 65% RH for seven days prior testing. The shear strength tests were performed under dry (ambient temperature) and wet (4 h in water at 23 ± 2 °C) measuring conditions.

### 2.7. Statistical Analysis

The results of tensile shear strength were analyzed with a one-way analysis of variance (ANOVA) at 0.05 significance level using the statistical software package IBM SPSS Statistics, Version 24 (IBM Corporation, New York, NY, USA), as described previously [23,24]. The significant differences between values were assessed by Duncan’s multiple-range test.

## 3. Results and Discussion

### 3.1. Thermal Behavior of the Adhesives

**Figure 1:**
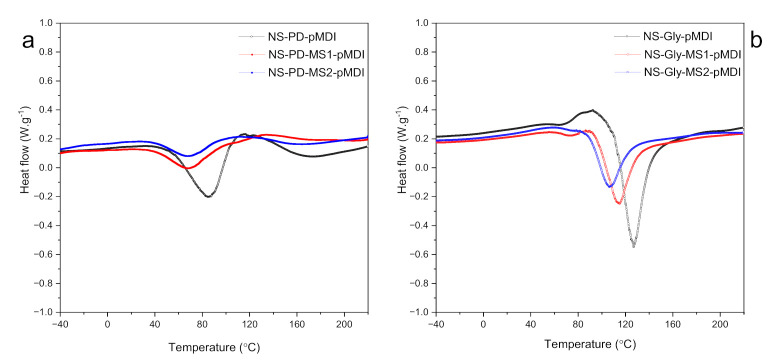
The first DSC thermographs of polyurethane adhesives with (**a**) NS, PD, MS, and pMDI, and (**b**) NS, Gly, MS, and pMDI.

The curing performance of the polyurethane adhesives was analyzed using a first differential scanning calorimetry (DSC) thermograph through a non-isothermal program (Figure 1a,b and Table 2). The curing process of thermosetting adhesives is usually accompanied by a significant change of heat, which is revealed as exothermic peaks in the DSC curve [20]. It should be noted that second scans were performed to ensure the complete curing of the adhesives, while no exothermic peak was observed. The exothermic curing peak, T_max_, of the control adhesive that contained PD (NS-PD-pMDI) was at 85.7 °C, while the formulation with Gly (NS-Gly-pMDI) exhibited a T_max_ of 127.2 °C. This might be related to the different reaction rates between isocyanate groups of pMDI and primary and secondary hydroxyl groups in the polyols [25]. It is known that the reaction of isocyanate groups with a primary hydroxyl group is about 3.3 times faster than with a secondary hydroxyl group [26]. PD contains two primary hydroxyl groups, while Gly contains two primaries and one secondary hydroxyl groups. Thus, more energy is required for Gly to perform the chemical reaction with pMDI than PD. The addition of MS lowered the curing temperatures. This was more pronounced in the adhesives that contained Gly. The respective T_max_ for NS-PD-MS1-pMDI and NS-PD-MS2-pMDI were 68.3 °C and 67.3 °C; however, the respective values for NS-Gly-MS1-pMDI and NS-Gly-MS2-pMDI were 114.5 °C and 106.7 °C. This might be explained by replacing the pMDI with functionality between two and three [27] with MS with more functionality (due to the existence of both –OH and –NCO groups in the anhydroglucose unit of MS), which could enhance the reaction rate [22]. A similar pattern was reported previously by adding a pre-polymer with NCO functionality to starch-based PUWAs [20]. The curing heats (∆H) of the NS-PD-pMDI and NS-Gly-pMDI adhesives were 91.5 J·g^−1^ and 83.1 J·g^−1^, respectively (Table 2). The curing heat, however, decreased considerably by increasing the MS content, and the trend was more pronounced in the adhesives with PD. The respective curing heats for NS-PD-MS1-pMDI and NS-PD-MS2-pMDI adhesives were 27.7 J·g^−1^ and 23.5 J·g^−1^, while NS-GLy-MS1-pMDI and NS-Gly-MS2-pMDI exhibited curing heat values of 74.7 J·g^−1^ and 50.6 J·g^−1^, respectively. This could be related to the decrease in the total equivalents of NCO or OH involved per gram of adhesive formulations.

**Figure 2:**
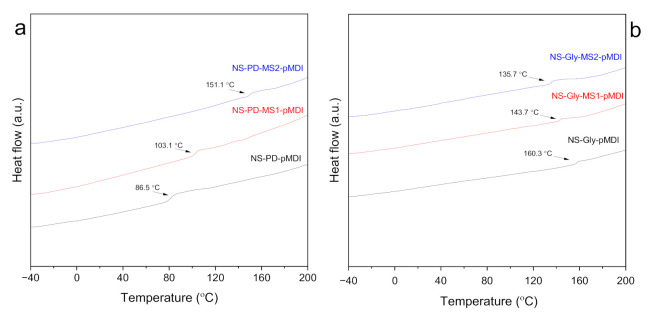
The DSC thermographs of cured adhesives with NS, PD, MS and pMDI (**a**) and with NS, Gly, MS and pMDI (**b**).

The Tg determination of the cured adhesives can provide information on the degree of polymer mixing and the miscibility of different components [28]. The Tg of the adhesives after removing the first history of samples are shown in Figure 2a,b. The application of different polyols (PD and Gly) in the presence of NS changed the Tg of the adhesives considerably. It should be noted that only one Tg was observed for each formulation, which indicates complete miscibility between the polymers [28]. The adhesive formulation prepared with NS-PD-pMDI exhibited a Tg at 86.5 °C, while the Tg in the NS-Gly-pMDI formulation was 160.3 °C. This might be related to a higher crosslinking density in the formulation prepared with trifunctional molecules than the ones with a diol [29]. The Tg of the adhesives increased by partial replacement of pMDI with MS when PD was used. However, Tg was slightly decreased in the formulations containing Gly. The NS-PD-MS1-pMDI and NS-PD-MS2-pMDI adhesives showed respective Tg values of 103.1 °C and 151.0 °C, while the respective values for NS-Gly-MS1-pMDI and NS-Gly-MS2-pMDI were 143.7 °C and 135.7 °C. The Tg alterations in the adhesives with a higher MS content can be ascribed to the different disposition of the –NCO groups in MS and pMDI and the content of components with different functionality, which results in different packaging. Symmetric aromatic diisocyanates such as pMDI have higher reactivity (higher Tg) than asymmetric aliphatic diisocyanates such as IPDI [26]. The effect of pMDI reduction on Tg in the formulations containing PD could be offset by the addition of a polymer (MS) with more functionality. Moreover, the replacement of pMDI with a polymer functionalized with isocyanate pendant groups, such as MS in our study, and in the presence of a short-chain polyol (PD), may restrict the molecular mobility of the PU chains, contributing to increasing the Tg of the system. This also suggests that the crosslinking density in the adhesive with PD polyol increased by increasing the MS content. The Tg decrease in the adhesives containing Gly polyol could not be attributed to a lower crosslinking density but to a mismatch of the chemical groups that are responsible for urethane bonding and to an increase or unevenness in the distance between urethane groups due to the addition of MS in the presence of lower Gly content [30]. The decrease in the Tg of Gly-containing adhesives is not as great as the increase in the Tg of PD systems, suggesting that the effect of the functionality of a small molecule such as Gly outweighs that of the substitution of pMDI with MS. These results are in agreement with the ones obtained in the thermomechanical analysis of polyurethane films with similar formulations [22].

**Figure 3:**
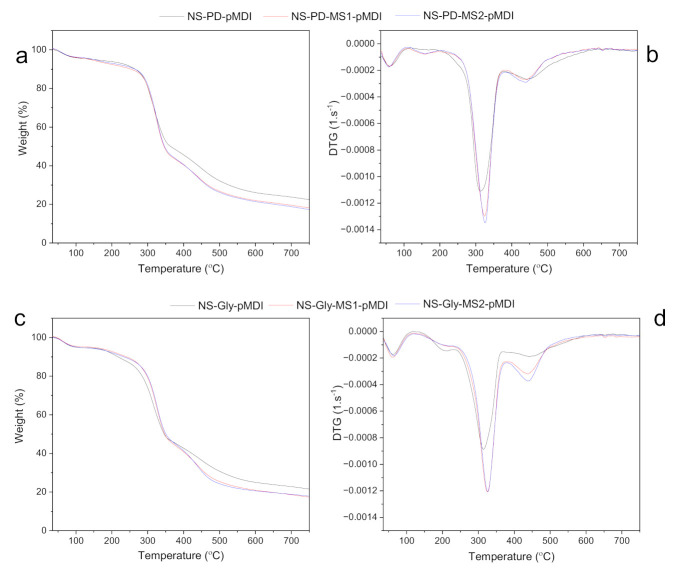
The weight loss and DTG curves of adhesives with NS, MS, PD, and pMDI (**a**,**b**), and with NS, MS, Gly, and pMDI (**c**,**d**).

The thermal degradation behavior of cured adhesives was examined by thermogravimetric (TG) and derivative thermogravimetric (DTG) analyses of the samples. The TG and DTG curves of the cured adhesives are shown in Figure 3a–d. There were slight differences in the degradation patterns of the adhesives as a function of polyols and MS. As can be seen from the TG figures (Figure 3a,c), the adhesives prepared without MS exhibited the highest residual weight (RW). NS-PD-pMDI and NS-Gly-pMDI showed RW values of 21.7% and 20.7%, respectively. However, the RW of the adhesives was lower in the presence of MS, as NS-PD-MS1-pMDI, NS-PD-MS2-pMDI, NS-Gly-MS1-pMDI, and NS-Gly-MS2-pMDI had RW values of 17.3%, 16.4%, 17.8%, and 17.6%, respectively. The decreased RW values of MS-containing adhesives could be related to the higher purity of MS because of its decontamination with different solvents during the modification process in comparison with NS. As can be seen from the DTG curves (Figure 3b,d) and the corresponding thermal degradation properties indicated in Table 3, various degradation patterns were obtained with different proportions of polymers in the adhesives, although the degradation behavior of some individual polymers could be overlapped. The first weight loss (T_max1_) peak occurred at temperatures below 100 °C, which could be related to the bounded water molecules in the structure of cured adhesive. This was followed by T_max2_ at ~156–157 °C for the adhesives containing PD and at ~215–218 °C for those containing Gly. The first stage of polyurethane degradation below 300 °C is due to the elimination of volatile components and the decomposition of biuret and allophanate linkages [31,32,33]. The T_max3_ peak shifted to slightly higher temperatures in the adhesives with MS. A diverse effect was observed for T_max4_, where the adhesives with MS showed somewhat lower T_max4_ than the ones without MS, which could be related to the thermolysis of the organic residues from previous steps [33]. In addition, the thermal degradation pattern at T_max3_ and T_max4_ peaks in the cured adhesives was similar to the ones reported previously for starch and dextrin polymers after modification with IPDI [15,22].

### 3.2. Rheological Performance

**Figure 4:**
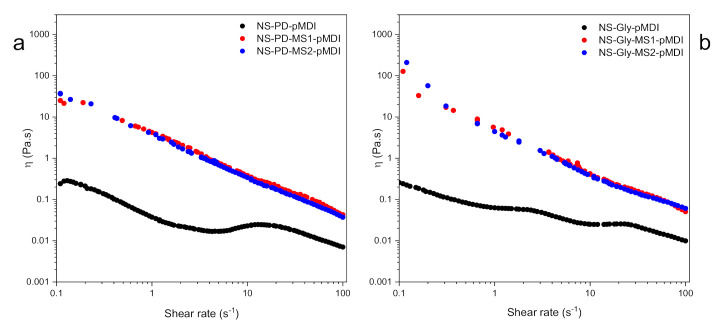
The dynamic rheological properties of polyurethane adhesives with (**a**) NS, PD, MS, and pMDI, and (**b**) NS, Gly, MS, and pMDI.

An adhesive’s application and performance are closely related to its rheological properties [34]. The viscosity change of the polyurethane adhesives with a logarithmic ascendant ramp for a shear rate from 0.1 to 100 s^−1^ at a constant temperature of 20 °C is shown in Figure 4a,b. Both NS-PD-pMDI and NS-Gly-pMDI adhesives exhibited a very low viscosity, as their zero shear viscosities (η_0_) were 0.037 Pa.s and 0.061 Pa.s, respectively. The lower the value of η, the stronger the de-structurization rate induced by the shear [35]. It was apparent that the addition of MS considerably increased the viscosity of the polyurethane adhesives, while no obvious differences were observed in the adhesives with higher MS content, e.g., NS-PD-MS2-pMDI and NS-Gly-MS2-pMDI. In this study, the rheological behavior of thermoset polyurethane adhesives was studied under room temperature, and thus, it is unlikely that a crosslinked network formed, as the adhesives were easily wiped off from the testing plates. At a comparable loading level of MS, the adhesives that contained Gly exhibited a higher viscosity than those with PD. This could be related to the formation of a more structured network. By increasing the shear rate, a shear thinning behavior was observed and the viscosity of the polyurethane adhesives containing MS linearly decreased with the shear rate in the log–log plot. The shear-thinning behavior facilitates the interaction between an adhesive and an adherend, e.g., wood, and higher viscosity suggests stronger interaction among the adhesive components [34], which may lead to a better bonding quality.

### 3.3. Tensile Shear Strength

**Figure 5:**
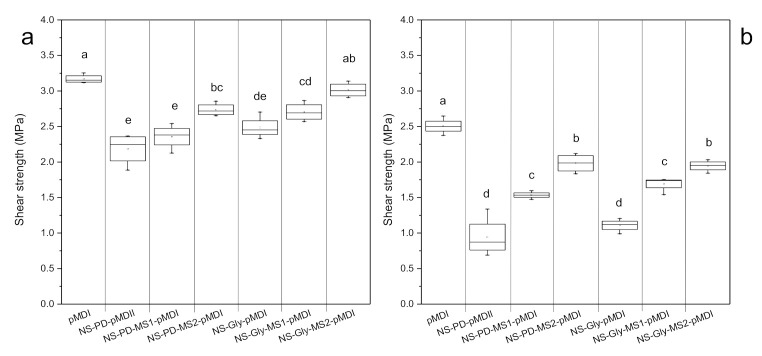
The tensile shear strength of veneers bonded with polyurethane adhesives. The substrates were evaluated under ambient conditions (dry, **a**) and after immersion in water for 4 h at 23 ± 2 °C (wet, **b**). In the boxplots with whiskers from the minimum to the maximum, the box illustrates the 25%, 50%, and 75% quartiles, and the mean value of each data set is depicted as quadrate inside the box. The values labelled with different letters are statistically different at an error probability of α = 0.05 (ANOVA and Tukey’s HSD tests), *n* = 10.

The tensile shear strength is a reliable test that is commonly used to evaluate the adhesion quality between two pieces of wood samples [16]. The shear strength of wood veneers bonded with polyurethane adhesives under dry and wet measuring conditions are presented in Figure 5a,b. For comparison reasons, one adhesive was prepared with only pMDI. It should be notified that the failures were accrued in wood veneers, which means that the adhesives were stronger than the veneers. The wood veneers bonded with NS-Gly-pMDI showed slightly higher strength than the ones with PD polyol under both dry and wet measuring conditions, although there were statistically insignificant differences (α = 0.05). The tensile shear strength of the wood veneers under both dry and wet conditions was considerable in adhesives containing MS. At a lower loading level of MS (MS1), the adhesives containing PD polyol showed inferior bonding performance compared with those containing Gly polyol. A similar trend was observed in the adhesives with MS2, while the differences were not statistically significant. The adhesive with Gly polyol and a high MS content (NS-Gly-MS2-pMDI) exhibited comparable tensile shear strength to the control samples (sole commercial pMDI) under the dry measuring condition (Figure 2a). As it was assumed from the rheological performances, the adhesives with a more structured network, e.g., in the presence of MS, and adequate level of shear-thinning behavior resulted in higher bonding strength. These results are in accordance with a previous study where the flexural bending strength of polyurethane films with a combination of MS and polyols was significantly higher than the ones without MS [22]. As described previously by Desai and coworkers [13], a higher crosslinking density of polyurethane adhesives with a high hydroxyl group content polyol leads to an improved bonding shear strength [35]. For all adhesives, the tensile shear strength of the wood veneers was decreased after 4 h of immersion in water. The penetration of water molecules into the wood structure acts as a plasticizer in the adhesive–wood interfaces, which results in the reduction in bonding strength under wet measuring conditions [34,36]. Although the control adhesive (sole commercial pMDI) showed superior strength in comparison to the other adhesives, the adhesives with MS illustrated some noticeable strength in the wet condition (Figure 3b).

## 4. Conclusions

Polyurethanes formulated with various combinations of bio-based and synthetic precursors were successfully applied as wood adhesives. The adhesives with Gly polyol showed a higher curing temperature and a slightly higher viscosity than the ones with PD. The addition of MS shifted the curing temperature to lower levels, altered the glass transition temperature, improved the thermal stability, increased the viscosity, and significantly contributed to the shear strength improvement of the adhesives with both polyol types. The adhesive with a higher load level of MS with Gly polyol (NS-Gly-MS2-pMDI) exhibited comparable tensile shear strength to the commercial pMDI adhesive under the dry measuring condition. This research showed that wheat starch functionalized with IPDI in combination with bio-based polymers (native wheat starch and glycerol) and a small amount of synthetic polymer (pMDI) has a high potential to be applied as a wood adhesive.

## Figures and Tables

**Table 1:** The adhesives with different combination levels (wt. %) of bio-based and synthetic polymers, adjusted from Hosseinpourpia et al. [22].

Adhesive	NS (wt. %)	Polyol (wt. %)	MS (wt. %)	pMDI (wt. %)
NS-PD-pMDI	35	13	-	52
NS-PD-MS1-pMDI	27	12	21	40
NS-PD-MS2-pMDI	25	10	29	36
NS-Gly-pMDI	35	11	-	54
NS-Gly-MS1-pMDI	28	9	22	41
NS-Gly-MS2-pMDI	26	8	30	36

MS1 and MS2 represent lower and higher load levels of MS, respectively. NS: native wheat starch, MS: modified starch, PD: 1,3-propanediol, Gly: glycerol.

**Table 2:** The details of the DSC thermograms of polyurethane adhesives.

Adhesive	T_max_ °C	∆H (J g^−1^)
NS-PD-pMDI	85.7	91.5
NS-PD-MS1-pMDI	68.3	27.7
NS-PD-MS2-pMDI	67.3	23.5
NS-Gly-pMDI	127.2	83.1
NS-Gly-MS1-pMDI	114.5	74.7
NS-Gly-MS2-Gly	106.7	50.6

**Table 3:** The thermal degradation properties of adhesives.

Adhesive	T_max1_	T_max2_	T_max3_	T_max4_	RW
	(°C)	(°C)	(°C)	(°C)	(%)
NS-PD-pMDI	60.2	157.2	311.8	442.0	21.7
NS-PD-MS1-pMDI	60.6	156.4	325.5	438.7	17.3
NS-PD-MS2-pMDI	58.4	156.1	326.9	438.1	16.4
NS-Gly-pMDI	64.7	215.9	315.6	443.0	20.7
NS-Gly-MS1-pMDI	62.0	216.9	325.4	427.6	17.8
NS-Gly-MS2-pMDI	64.5	217.6	326.1	439.5	17.6

## Data Availability

The data presented in this study are available from the corresponding author upon request.
